# Emerging strategies in senotherapeutics: from broad-spectrum senolysis to precision reprogramming

**DOI:** 10.1038/s41514-026-00355-z

**Published:** 2026-03-10

**Authors:** Weidong Zhang, Shijie Song, Yue Zhang, Yong Pan, Dahai Hu, Yunchuan Wang

**Affiliations:** 1 Department of Burns and Cutaneous Surgery, Xijing Hospital, Xi’an, China; 2 Xi’an Helenzone Plastic and Cosmetic Clinic, Xi’an, China

**Keywords:** Cancer, Cell biology, Drug discovery, Immunology

## Abstract

Cellular senescence, originally described as a finite proliferative arrest in cultured somatic cells, has since been recognized as a central mechanism underlying aging and the development of age-associated disorders. The progressive accumulation of senescent cells (SnCs) promotes chronic inflammation through the senescence-associated secretory phenotype (SASP) and circumvents immune-mediated clearance by upregulating pro-survival and immune checkpoint pathways. Early “first-generation” senolytics, including navitoclax (ABT-263) and the dasatinib–quercetin (D + Q) combination, provided proof-of-concept that selective removal of SnCs can alleviate certain fibrotic, metabolic, and cardiovascular pathologies in preclinical studies. However, these agents exhibited notable drawbacks, such as dose-dependent thrombocytopenia, variable therapeutic efficacy, and the emergence of resistance mechanisms. Consequently, current research has shifted toward precision senotherapy, though significant translational challenges remain. This review synthesizes three next-generation strategies developed to address limitations of early senolytic agents. (1) Immune-based senolysis: This approach applies immuno-oncology principles to counter immune evasion of SnCs. Strategies include blocking immunosuppressive ligands such as GD3 ganglioside, engineering chimeric antigen receptor (CAR) T cells to target senescence-specific surface markers like urokinase-type plasminogen activator receptor (uPAR), and exploiting metabolic vulnerabilities (e.g., glutaminolysis and ferroptosis) to sensitize SnCs to immune-mediated clearance. (2) Tissue-precision proteolysis-targeting chimeras (PROTACs): These agents recruit organ- or tissue-specific E3 ligases (e.g., von Hippel-Lindau (VHL)) to selectively degrade anti-apoptotic proteins such as BCL-xL. Localized activity may reduce systemic toxicity and mitigate dose-limiting effects observed with traditional inhibitors. (3) Microbiome–epigenetic interplay: This strategy modulates the gut–liver axis to enhance senolytic efficacy. Short-chain fatty acids (SCFAs), such as butyrate, epigenetically regulate drug transporter expression and suppress the SASP, while dietary interventions may create a microenvironment favorable to senolysis. These approaches offer potentially more targeted and personalized therapeutic options but face significant challenges, including immunopathology, manufacturing complexity, off-target effects, and long-term safety concerns. The ongoing shift from broad inhibition to precision reprogramming represents a promising but preliminary step in the treatment of age-related diseases.

## Introduction

### Evolution of senescent cell (SnC) research

In the 1960s, Leonard Hayflick’s discovery that cultured human fibroblasts cease dividing after a finite number of population doublings—termed the “Hayflick limit”—established the foundation of cellular senescence research^1^. This finding refuted Nobel laureate Alexis Carrel’s long-held belief that normal cells were immortal in culture. Hayflick and Moorhead showed that normal human fetal cells divide only 40–60 times before entering irreversible growth arrest. Initially met with skepticism, this discovery became a cornerstone of modern aging biology.

Originally regarded as a replicative safeguard against malignant transformation^2^, cellular senescence is now recognized as a multifaceted and dynamic stress response. It can be induced by various triggers, including telomere shortening, oncogenic signaling, oxidative stress^3^, genomic instability, DNA damage^4^, and mitochondrial dysfunction^5^. This conceptual evolution has redefined senescence as a double-edged phenomenon: while it acts as a potent tumor suppressor and contributes positively to processes such as wound repair and embryonic development, the chronic accumulation of SnCs promotes aging and age-related disorders. This paradox exemplifies the evolutionary principle of antagonistic pleiotropy, which suggests that genes providing reproductive or survival advantages early in life may persist even if they exert harmful effects later, when selective pressures decline^6^. Thus, senescence confers an early-life benefit by halting the proliferation of potentially oncogenic cells, yet in later life, the persistence of these same cells drives chronic inflammation and functional tissue deterioration.

SnCs possess strong anti-apoptotic mechanisms, primarily driven by the upregulation of BCL-2 family proteins, enabling them to resist programmed cell death^7,8^^.^ In addition to upregulating anti-apoptotic pathways, they evade immune surveillance by modulating immune checkpoints such as GD3 ganglioside^9^ and secrete a pro-inflammatory mixture of cytokines, chemokines, and proteases collectively termed the senescence-associated secretory phenotype (SASP)^10,11^. Moreover, emerging evidence underscores the remarkable heterogeneity of SnCs, characterized by distinct molecular profiles, such as p16INK4A-high and p21CIP1-high phenotypes, across different tissues and pathological conditions. This diversity emphasizes the need for precision-based interventions capable of selectively eliminating or reprogramming deleterious SnCs while preserving their beneficial physiological functions^12,13^. Despite promising preclinical data, clinical translation of senotherapeutics remains hindered by SnC heterogeneity, off-target toxicity, variable efficacy across models, and lack of robust biomarkers.

### SnC accumulation and age-associated pathologies

The potential translational relevance of senescent cell (SnC) biology has expanded as evidence links their accumulation to diverse chronic diseases. SnCs disrupt tissue homeostasis through sustained cell-cycle arrest and the pro-inflammatory activity of the SASP. Notable examples include: (1) Fibrotic disorders: In idiopathic pulmonary fibrosis (IPF), SnCs secrete profibrotic mediators such as transforming growth factor-beta (TGF-β), promoting excessive extracellular matrix deposition and lung scarring^14^. (2) Osteoarthritis: Senescent chondrocytes release catabolic enzymes, particularly matrix metalloproteinase-13 (MMP-13), leading to cartilage degradation and persistent joint inflammation^15^. (3) Neurodegeneration: Senescent glial cells enhance neuroinflammation through SASP factors, accelerating neuronal loss in Alzheimer’s and Parkinson’s diseases^16,17^. (4) Cardiovascular diseases: Senescent endothelial and foam cells contribute to vascular dysfunction, endothelial barrier impairment, and atherosclerotic plaque destabilization^18,19^. Intermittent senolytic treatment has also been shown to restore metabolic health in some models of obesity and type 2 diabetes^20^.

The concept of senolytics—agents that selectively eliminate SnCs—emerged from early studies using navitoclax (ABT-263) and the dasatinib plus quercetin (D + Q) combination. These first-generation senolytics target SnC anti-apoptotic pathways (SCAPs), which enable resistance to apoptosis despite SASP-induced stress^8^. While these agents validated the therapeutic potential of SnC clearance in certain contexts, they revealed major limitations, including dose-dependent toxicity, limited efficacy in fibrotic tissues, resistance mechanisms such as MCL-1 upregulation, and negative outcomes in some models^21^. These issues have driven the development of next-generation precision senotherapeutics.

### Scope of this review

**Fig. 1:**
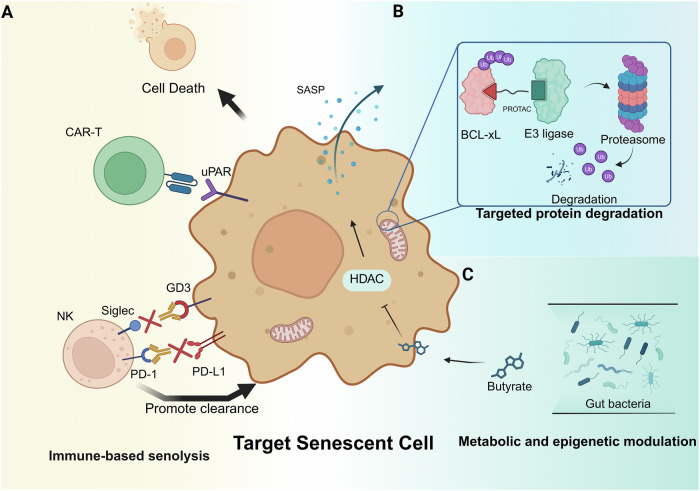
A synergistic framework illustrating next-generation senotherapeutic strategies. A central pathological senescent cell displays multiple defense mechanisms, including anti-apoptotic proteins (BCL-xL), secretion of a pro-inflammatory SASP, and surface receptors for immune evasion (uPAR, GD3 Ganglioside). Three synergistic therapeutic modules are depicted targeting these defenses: **A**. *Immune-based senolysis*, utilizing engineered immune cells (e.g., *CAR-T cells, shown top left*) and checkpoint blockade antibodies to overcome immune tolerance and clear senescent cells. **B** *Targeted protein degradation*, employing PROTACs to recruit VHL E3 ligase, leading to the ubiquitination and subsequent proteasomal degradation of BCL-xL. **C** *Metabolic and epigenetic modulation*, where gut microbiota-derived metabolites like butyrate enter circulation and can suppress the SASP via HDAC inhibition, while potentially modulating drug transporters to enhance therapeutic synergy. Figure created with BioRender.com.

This review examines the mechanisms, achievements, and limitations of first-generation senolytics, followed by an exploration of three emerging next-generation strategies designed to address their shortcomings. We discuss that forthcoming advancements in senotherapeutics may extend beyond single-pathway inhibition, potentially emphasizing the integration of immune modulation, targeted protein degradation, and metabolic reprogramming. These approaches represent potentially more refined and durable interventions but also introduce new challenges, including immunopathology, manufacturing complexities, off-target effects, and the need for highly personalized design. Despite these hurdles, they represent a promising area in aging research, though clinical evidence remains preliminary (Fig. 1).

## Early senolytic interventions

### The rationale behind first-generation senolytics

**Fig. 2:**
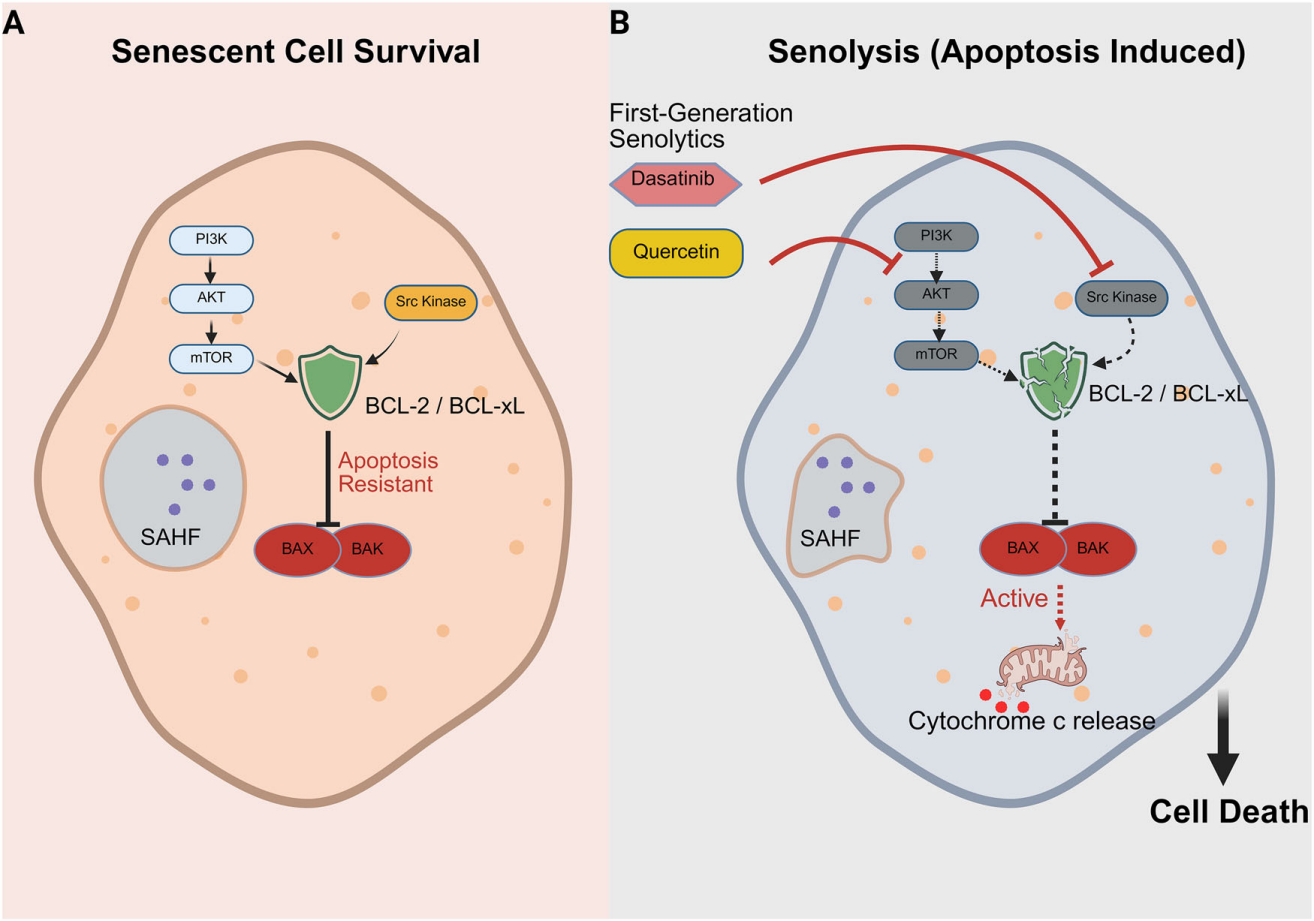
Contrasting survival and apoptosis in senescent cells targeted by first-generation senolytics. **A** The survival state of a senescent cell is maintained by active anti-apoptotic pathways. Pro-survival signals from pathways involving PI3K and Src Kinase support the function of BCL-2 family proteins (BCL-xL, BCL-2), which act to inhibit the pro-apoptotic proteins BAX/BAK. This robust defense mechanism makes the cell resistant to apoptosis. The nucleus contains senescence-associated heterochromatin foci (SAHF). **B** *First-generation senolytics*, such as the Dasatinib and Quercetin (D + Q) cocktail, induce apoptosis by disabling these survival pathways. This leads to the neutralization of BCL-xL and BCL-2, allowing BAX/BAK to become active, permeabilize the mitochondria, and trigger the apoptotic cascade, evidenced by membrane blebbing and cellular fragmentation. Figure created with BioRender.com.

First-generation senolytics act by targeting proposed senescence-associated anti-apoptotic pathways (SCAPs), molecular networks that SnCs may upregulate to withstand pro-inflammatory and pro-apoptotic stress potentially induced by their own SASP (though direct cell-autonomous effects remain under investigation)^22^. Central targets include BCL-2 family proteins (such as BCL-xL and BCL-2) and pro-survival kinase cascades (including Src and PI3K/AKT)^23–25^. Inhibiting these SCAPs disrupts the survival machinery of SnCs, triggering their selective apoptosis while sparing normal cells in some contexts^26,27^ (Fig. 2). The principal classes include: (1) BCL-2 Family Inhibitors: Navitoclax (ABT-263), ABT-737; (2) Kinase Inhibitors: Dasatinib; and (3) Flavonoids/Polyphenols: Quercetin, Fisetin^28^.

### Representative agents in greater detail

#### Navitoclax (ABT-263)

Navitoclax functions as a potent inhibitor of BCL-xL, BCL-2, and BCL-w. In aged murine models, treatment restored hematopoietic stem cell function^29^ and reversed radiation-induced tissue damage^30,31^. Despite preclinical outcomes, clinical applicability is limited by dose-dependent thrombocytopenia, as platelets rely on BCL-xL^32^. In oncology trials, 44–50% of patients experienced grade 3 or 4 thrombocytopenia^33,34^. This on-target toxicity highlights a key limitation of first-generation senolytics.

#### D + Q

The D + Q combination targets multiple SCAPs. In aged murine models, intermittent administration improved physical function and extended lifespan in some studies^35^. In a pilot study in diabetic kidney disease (NCT02848131), a 3-day course reduced SnC burden in adipose tissue^20^. An early-phase pilot study in IPF suggested modest functional trends^36^, and a Phase 2 trial in postmenopausal women showed limited biomarker changes with subtle or no significant clinical improvements in bone mass^37^. However, the efficacy of D + Q and other senolytics has shown variability across models and contexts, with inconsistent effects on senescence markers and limited evidence of lifespan extension in more rigorous or genetically diverse preclinical settings^38^, underscoring potential influences of dosing, genetic background, or model differences^27^. Broader utility is constrained by poor tissue penetration, low bioavailability, and context-dependent efficacy (e.g., worsening outcomes in acute kidney injury models)^39^.

#### Natural Polyphenols (Fisetin)

Fisetin targets BCL-xL and PI3K/Akt/mTOR pathways. Comparative studies showed greater potency than quercetin in some models^40,41^. However, results from the NIA Interventions Testing Program in genetically heterogeneous UM-HET3 mice showed no significant extension of median or maximum lifespan with fisetin treatment (600 ppm), highlighting potential discrepancies due to dosing, timing, genetic background, or strain differences^42^. Ongoing human trials are evaluating fisetin in frailty and other conditions (Table 1). A novel galactomannan formulation from fenugreek seeds enhanced bioavailability up to 27-fold in a human study^43^, representing a potential improvement for clinical translation, though no galactomannan-based formulations are currently approved for oral medications.

**Table 1:** Selected clinical trials of senolytics

Drug/Combination	Indication/Condition	Phase	ClinicalTrials.gov ID (NCT)	Status/Key Outcomes	Limitations
Dasatinib + Quercetin (D + Q)	Diabetic kidney disease	Pilot	NCT02848131	Reduced SnC burden in adipose tissue	Small sample size, short-term follow-up
Dasatinib + Quercetin (D + Q)	Idiopathic pulmonary fibrosis (IPF)	Pilot	NCT02874989	Modest trends in functional improvements	Small sample size, inconclusive results
Dasatinib + Quercetin (D + Q)	Bone health in postmenopausal women	Phase 2	Farr et al.^37^ NCT04313634	Limited biomarker changes; subtle or no clinical benefits	Lack of significant clinical improvements
Fisetin	Frailty in cancer survivors	Phase 2	NCT05595499; NCT06113016	Ongoing	Early stage; bioavailability concerns
Fisetin	Peripheral artery disease	Pilot	NCT06399809; NCT06133634	Ongoing	Preliminary; limited data

#### Other exploratory agents

Beyond the more established senolytics, several preclinical strategies target distinct vulnerabilities in SnCs. The FOXO4-DRI peptide disrupts the FOXO4–p53 interaction, promoting selective apoptosis in SnCs^44–46^. Piperlongumine elevates reactive oxygen species (ROS) levels, exploiting the heightened oxidative stress susceptibility of SnCs^47^. Cardiac glycosides, such as ouabain and digoxin, inhibit Na⁺/K⁺-ATPase and may capitalize on altered membrane potential or reduced stress tolerance that is more pronounced in SnCs than in non-SnCs^48,49^. Additionally, SnCs exhibit increased reliance on autophagy for survival; autophagy inhibitors, such as chloroquine and bafilomycin A1, have been reported to selectively induce apoptosis by disrupting lysosomal function and autophagic flux, highlighting a potential metabolic vulnerability^50,51^. Specifically, chloroquine has demonstrated senolytic activity in therapy-induced senescent glioblastoma cells by blocking autophagosome-lysosome fusion and reducing senescence markers^50^, while bafilomycin A1 disrupts cytoprotective autophagy in SnCs, leading to accumulation of damaged mitochondria and apoptotic cell death^51^. These approaches remain largely exploratory, with evidence primarily derived from in vitro and specific in vivo models, and further validation is needed for clinical translation.

### The drive for precision

First-generation senolytics provided critical proof-of-concept for the therapeutic potential of SnC clearance^52^. Nevertheless, their clinical translation is hampered by limited specificity, resulting in on-target, off-tissue toxicities^53^. In addition, incomplete efficacy and the capacity of SnCs to evade elimination by upregulating alternative survival pathways (such as MCL-1)^54,55^ or modulating immune checkpoints underscore the need for more sophisticated therapeutic approaches^56^. Negative results in heterogeneous models and certain clinical contexts further highlight the complexity of the field. These limitations have catalyzed the development of next-generation strategies aimed at achieving greater precision, potency, and durability in senolytic interventions.

## Immune-based senolysis

A central factor contributing to the accumulation of SnCs is their ability to evade immune-mediated clearance. Interestingly, the mechanisms underlying this immune escape closely mirror those utilized by tumor cells, offering valuable insights and a conceptual framework for the development of immune-based senotherapeutic strategies^57,58^.

### Overcoming SnC immune evasion

SnCs actively establish an immunosuppressive microenvironment. Recent research has identified GD3 ganglioside as a pivotal “senescence immune checkpoint”. GD3 is upregulated on the surface of SnCs in aging tissues and engages inhibitory Siglec receptors (e.g., Siglec-7) on natural killer (NK) cells, thereby suppressing their cytotoxic function. In murine models of fibrosis, anti-GD3 immunotherapy disrupted this immune tolerance, resulting in a marked reduction of SnC burden and improved tissue function^9^. However, as with other immune checkpoint therapies, targeting this pathway carries a risk of autoimmunity, particularly in older individuals with pre-existing immune dysregulation^59,60^. Additionally, SnCs express other checkpoint ligands, including PD-L1 and HLA-E, which inhibit T-cell and NK-cell activity, respectively^61,62^. These findings suggest that multi-checkpoint blockade may offer a potent strategy to restore effective immune surveillance and clearance of SnCs, though risks of immunopathology must be carefully managed.

### Senolytic CAR-T cell therapy: a “living drug” approach

**Fig. 3:**
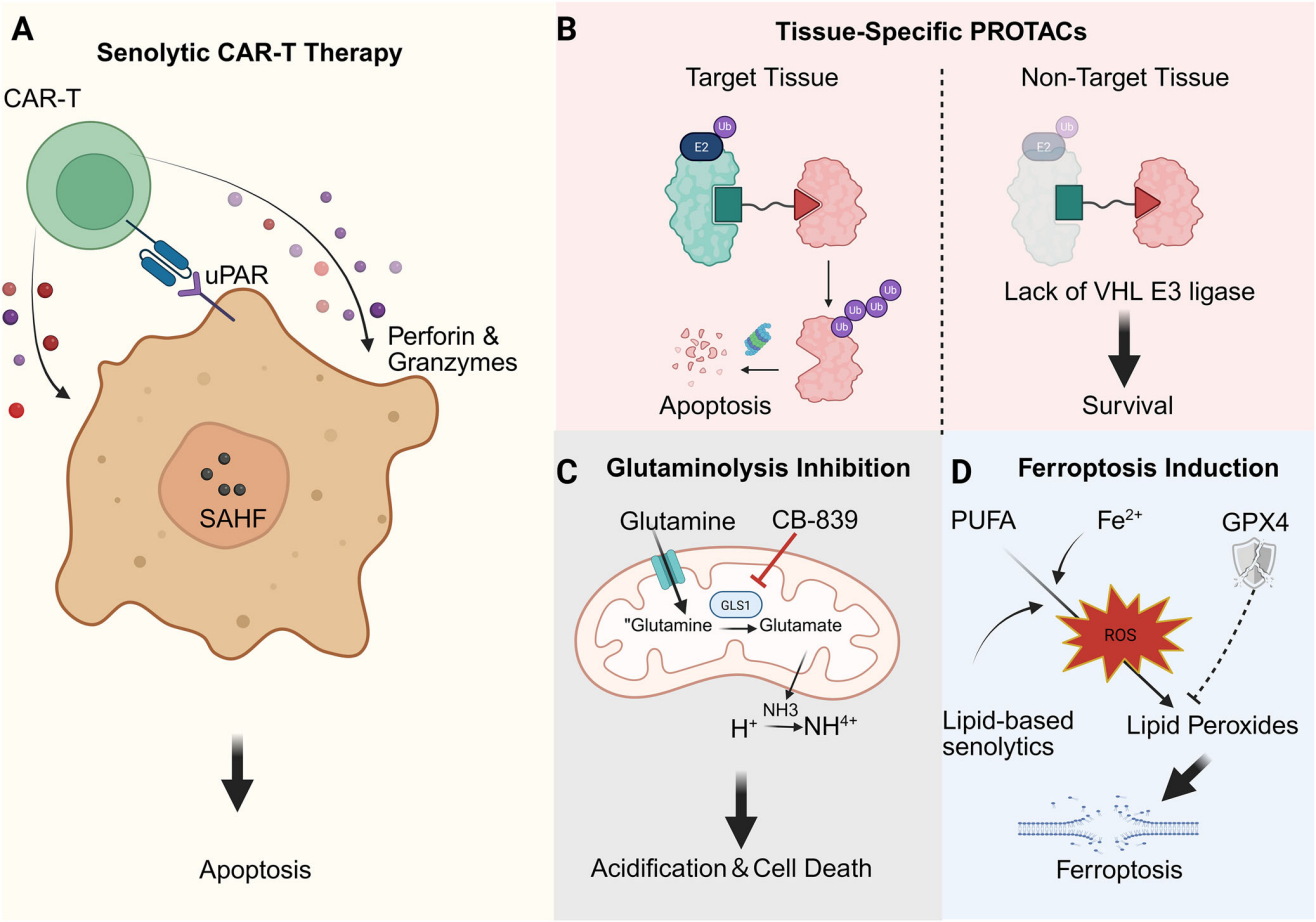
Key molecular mechanisms of action for emerging precision senotherapeutics. a, Senolytic CAR-T Therapy. **A** *Senolytic CAR-T Therapy*. The chimeric antigen receptor (CAR) on an engineered T cell specifically recognizes and binds to a surface antigen (uPAR) on a senescent cell. This binding activates the T cell to release cytotoxic granules containing perforin and granzymes to initiate apoptosis. **B** *Tissue-Specific PROTACs*. In a target tissue like the liver with high VHL expression, a PROTAC efficiently forms a ternary complex to degrade BCL-xL, causing apoptosis. In a non-target tissue (e.g., platelets) with lack of VHL E3 ligase, this process cannot occur, sparing BCL-xL and preventing toxicity. **C** *Glutaminolysis Inhibition*. Senescent cells with cytoplasmic acidosis depend on the enzyme GLS1 to produce ammonia (NH₃) for neutralization. A GLS1 inhibitor (e.g., *CB-839*) blocks this metabolic adaptation, leading to lethal intracellular acidification and cell death. **D** *Ferroptosis Induction*. A ferroptosis-inducing agent (e.g., *lipid-based senolytics*) exploits the inherent vulnerabilities of senescent cells—iron overload and high levels of polyunsaturated fatty acids (PUFAs) coupled with low GPX4 activity—to trigger runaway lipid peroxidation, resulting in catastrophic membrane rupture. Figure created with BioRender.com.

The most advanced application of immunotherapy in senolysis is the development of CAR T-cells. By engineering a patient’s T-cells to specifically recognize senescence-associated surface antigens, CAR-T therapy achieves highly precise targeting of SnCs. Genome-wide CRISPR screens and transcriptomic profiling have identified several promising SnC-specific targets, with urokinase-type plasminogen activator receptor (uPAR) emerging as a leading candidate^63,64^ (Fig. 3A). In seminal preclinical studies, a single dose of uPAR-targeted CAR T-cells was sufficient to reverse liver fibrosis, restore intestinal stem cell function, and confer long-term metabolic benefits in aged mice^65^. Additional targets under investigation include NKG2D ligands and DPP4, expanding the potential repertoire for CAR-T–based senolytic therapies^66^.

To enhance safety, researchers are developing logic-gated CAR constructs that require recognition of multiple senescence-specific antigens for activation (“AND” gate) or deliver inhibitory signals upon detection of healthy-cell antigens (“NOT” gate), thereby minimizing off-target cytotoxicity^67^. Despite these advances, major translational barriers remain, including high CAR-T production costs, risks of severe adverse events such as cytokine release syndrome (CRS), and reduced T-cell efficacy in older patients due to immunosenescence^68,69^. Emerging evidence links CAR-T cell aging to therapeutic failure, identifying cellular senescence as a primary mechanism of relapse. Interventions such as NAD⁺ supplementation and costimulatory domain optimization have been proposed to enhance T-cell durability and prevent dysfunction^70,71^.

### Exploiting metabolic vulnerabilities

Beyond direct immune targeting, a promising approach is to exploit the unique metabolic vulnerabilities of SnCs to selectively induce cell death: (1) Glutaminolysis Dependence: SnCs often exhibit lysosomal membrane damage, resulting in reduced intracellular pH (cytoplasmic acidification). To maintain survival, they upregulate glutaminase 1 (GLS1), which converts glutamine into glutamate and ammonia, helping neutralize excess acidity. This creates a critical metabolic dependency, and GLS1 inhibitors such as BPTES or CB-839 have been shown to selectively induce lethal acidification in a broad spectrum of SnCs^72,73^ (Fig. 3C). (2) Ferroptosis Sensitization: Ferroptosis, an iron-dependent form of programmed cell death driven by lipid peroxidation, exploits intrinsic vulnerabilities of SnCs. These cells accumulate high levels of iron and polyunsaturated fatty acids while exhibiting reduced expression of glutathione peroxidase 4 (GPX4), the key negative regulator of ferroptosis^74^. Although SnCs can develop resistance mechanisms, such as ferritin-mediated iron sequestration or lysosomal dysfunction impairing cystine transport^75^, these barriers can be overcome. Recent studies have identified lipid-based senolytics, including α-eleostearic acid, that selectively trigger ferroptosis in SnCs, lowering senescence burden and extending healthspan in mice^76,77^. This strategy represents a novel mechanistic avenue for senolysis, distinct from conventional apoptosis-based approaches, with emerging evidence showing that senescent macrophages can induce ferroptosis in skeletal muscle during osteoarthritis progression^78^ (Fig. 3D). These approaches may theoretically offer greater selectivity compared to first-generation senolytics, but in vivo studies have not yet rigorously assessed off-target effects with appropriate controls, and resistance mechanisms remain a concern^79^.

## Tissue-precision PROTACs

### Fundamentals of PROTAC-based senolysis

PROTACs constitute a transformative therapeutic platform that overcomes key limitations of conventional small-molecule inhibitors. These bifunctional molecules function as molecular matchmakers, simultaneously binding a target protein (e.g., BCL-xL) and an E3 ubiquitin ligase^80^. This induced proximity promotes ubiquitination of the target protein, directing it to the proteasome for complete degradation^81–83^. Unlike traditional inhibitors, which merely block protein activity, PROTACs remove the protein entirely, conferring several distinct advantages: (1) Complete Protein Removal: Eliminates the target protein entirely, preventing compensatory mechanisms and overcoming resistance associated with target overexpression; (2) Catalytic Activity: A single PROTAC molecule can mediate the degradation of multiple target proteins, enabling higher potency at lower doses; (3) Enhanced Specificity: By recruiting E3 ligases with tissue-restricted expression, PROTACs can achieve organ- or tissue-specific protein degradation, minimizing systemic toxicity^84–86^.

### Overcoming off-target toxicity with von Hippel-Lindau (VHL)-based PROTACs

The promise of PROTACs for precision senolysis is exemplified by strategies designed to mitigate the platelet toxicity associated with navitoclax. Researchers have engineered PROTACs that target BCL-xL for degradation by recruiting the VHL E3 ligase, which is widely expressed in many tissues but minimally present in platelets^85^. This tissue-selective degradation was first demonstrated using DT2216, a VHL-recruiting BCL-xL PROTAC that achieved potent antitumor activity without the severe thrombocytopenia observed with navitoclax^87^ (Fig. 3B).

Building upon this foundation, recent studies have developed next-generation dual-targeting degraders. In a landmark preclinical study published in Nature Aging, a VHL-based dual BCL-xL/BCL-2 PROTAC named 753b demonstrated potent, liver-tropic senolytic activity. In mouse models of non-alcoholic steatohepatitis (NASH), 753b efficiently cleared senescent hepatocytes and reduced liver fibrosis by over 50% without causing significant thrombocytopenia at the assessed time points^88^. However, experience with earlier VHL-based PROTACs suggests caution: DT2216-treated mice showed rapid platelet rebound following drug withdrawal^89^, indicating that detailed time-course analyses with parallel thrombocytopenic comparators (e.g., navitoclax) remain necessary to exclude transient hematologic effects with newer agents like 753b.

### Confronting resistance and enhancing delivery

SnCs can escape single-target senolytics by upregulating alternative pro-survival proteins, such as MCL-1. To address this, dual-targeting PROTACs capable of degrading both BCL-xL and BCL-2 have been developed, demonstrating high efficacy in models of therapy-induced senescence^90,91^. To further enhance tissue specificity, PROTACs can be coupled to liver-targeting moieties. For instance, Liver-Targeting Chimeras (LIVTACs) conjugated with triantennary N-acetylgalactosamine (tri-GalNAc) enable selective delivery to hepatocytes via the asialoglycoprotein receptor (ASGPR)–mediated endocytosis, increasing local drug concentration while reducing systemic exposure and “on-target off-tissue” toxicities such as thrombocytopenia^92^. This strategy has been successfully applied to degrade intracellular targets like BRD4 in hepatic stellate cells, reversing hepatic fibrosis in murine models^93^, and to target hepatocellular carcinoma through cathepsin B–responsive linkers^94^. Recent innovations also include nanoparticle-based delivery systems, which improve bioavailability and limit off-target effects in other VHL-expressing tissues^95^. Despite these advances, off-target degradation in non-target VHL-expressing tissues remains a potential concern, necessitating careful optimization of dosing strategies and linker chemistry^96^.

## Microbiome–epigenetic interplay

The gut microbiome has emerged as a key regulator of host metabolism and immune function, carrying significant implications for senotherapeutic strategies^97^. In particular, the gut–liver axis represents a promising target for improving both the efficacy and safety of senolytic interventions. Recent global research underscores the bidirectional interactions between gut microbiota and cellular senescence, with microbiome-focused therapeutics demonstrating potential to promote healthier aging^98^.

### SCFAs as epigenetic and metabolic modulators

Gut microbiota metabolize dietary fibers into SCFAs, including acetate, propionate, and butyrate. Butyrate functions as a potent histone deacetylase (HDAC) inhibitor, capable of epigenetically regulating gene expression in the intestinal epithelium and liver, thereby modulating systemic metabolic homeostasis^99^. Moreover, butyrate suppresses NF-κB activation and downregulates mTOR signaling, thereby reducing pro-inflammatory SASP cytokine secretion (e.g., IL-6, IL-8, and IL-1β) by approximately 40–50% in aged T cells in vitro^100^. Through the gut–liver axis, microbiota-derived metabolites including butyrate may indirectly influence the hepatic microenvironment, potentially enhancing the efficacy of senolytic interventions by attenuating chronic inflammation and creating a metabolic state less permissive to cellular senescence^101^.

### Dietary interventions and other metabolites

Omega-3 fatty acids (EPA and DHA) are widely recognized for their anti-inflammatory effects, primarily mediated through inhibition of NF-κB signaling^102,103^. When administered alongside senolytic therapies, they may modulate the transient inflammatory responses associated with SnC clearance^104^. Rather than suppressing the regenerative potential of this response, Omega-3 fatty acids may facilitate its timely resolution, preventing maladaptive chronic inflammation and thereby fostering a microenvironment more favorable for tissue repair. Additional microbial metabolites, such as conjugated bile acids, can activate the farnesoid X receptor, suppressing hepatic stellate cell activation and fibrogenic signaling, thereby ameliorating liver fibrosis^105,106^. Recent studies have also mapped age-related shifts in gut microbiota, revealing functional changes that modulate cellular senescence through intertwined metabolic and immune pathways^107,108^.

### Clinical translation and personalized approaches

Translating these insights into clinical practice is challenging due to substantial inter-individual variability in gut microbiome composition. Personalized strategies, such as microbiome profiling using 16S rRNA sequencing followed by customized prebiotic or probiotic interventions, may be necessary to standardize SCFA production and maximize senolytic efficacy. Preclinical data suggest that stable microbiome alterations, typically achievable within 2–4 weeks of dietary intervention, are required to produce consistent effects^109^. Moreover, emerging studies examining the impact of senotherapeutic agents on the human gut microbiota highlight the potential of microbiome-targeted interventions to prevent or mitigate age-related diseases. Long-term rejuvenation strategies have been shown to enhance organismal function, emphasizing the critical role of gut health in cardiovascular protection and overall healthy aging^110,111^. However, inter-individual variability and the need for sustained interventions pose significant hurdles.

## Additional emerging synergies and innovative features

### Comparing next-generation senolytics with conventional methods

Next-generation senolytics may represent advances over first-generation agents, potentially offering enhanced selectivity, efficacy, and safety in selected models (Table 2). Immunotherapies, such as CAR-T cells, provide highly specific antigen-targeted clearance, while PROTACs allow for tissue-specific degradation of intracellular proteins. Collectively, these strategies may overcome some limitations of early senolytics, which were frequently hampered by off-target toxicities and incomplete efficacy arising from the molecular heterogeneity of SnCs^2^. However, new risks and limited direct comparisons underscore ongoing challenges.

**Table 2:** Comparison of first-generation and next-generation senolytics

Aspect	First-Generation Senolytics (e.g., Navitoclax, D + Q)	Next-Generation Senolytics (e.g., CAR-T, PROTACs)
Selectivity	Moderate; targets broad SCAPs shared with healthy cells	Potentially higher; tissue-specific or antigen-targeted in selected models
Efficacy	Variable; partial SnC reduction in preclinical models	Potentially improved in targeted preclinical models
Toxicity	High risk of on-target, off-tissue effects (e.g., thrombocytopenia)	Lower systemic toxicity but risk of immunopathology (e.g., CRS)
Resistance	Common due to upregulation of alternative pathways (e.g., MCL-1)	Can be partially addressed via multi-targeting or logic-gated designs
Clinical Stage (2025)	Phase II trials ongoing for multiple indications	Preclinical to early Phase I

Comparisons are qualitative due to heterogeneity in preclinical models, tissues, endpoints, and measured SnC types; direct head-to-head studies are limited.

### A proposed framework for personalized senolysis

**Fig. 4:**
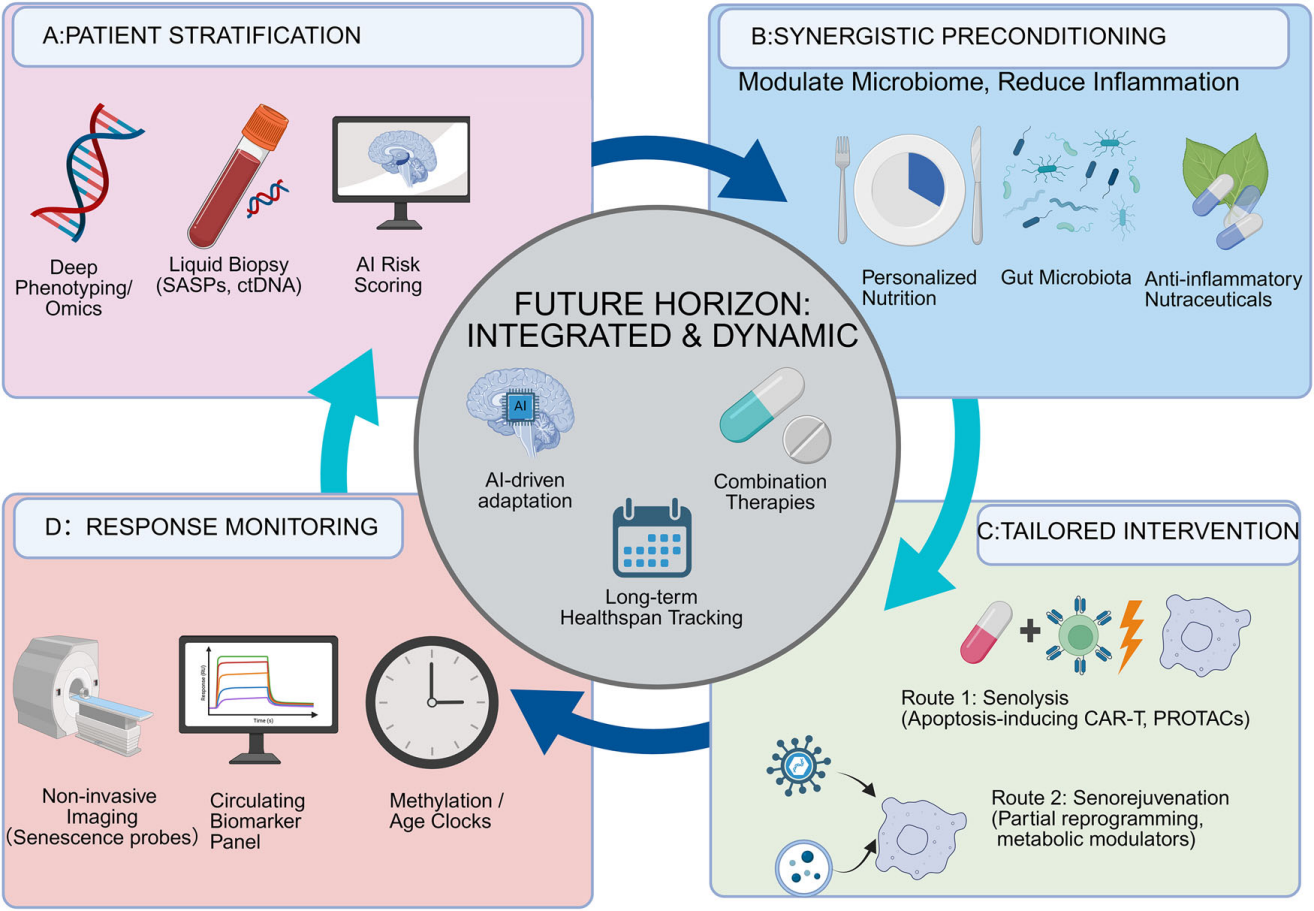
A proposed workflow for the clinical application of personalized senotherapy. This schematic illustrates a cyclical, biomarker-driven approach to treatment. **A** *Patient Stratification & Biomarkers*: Patients are first profiled using non-invasive biomarkers like PET imaging, liquid biopsies, and AI-driven risk scoring to quantify senescent cell burden and identify molecular dependencies. **B** *Synergistic Preconditioning*: A preparatory phase (2–4 weeks), utilizing strategies such as microbiome modulation and anti-inflammatory nutraceuticals, is employed to optimize the patient’s physiological state for therapy. **C** *Tailored Intervention*: Based on the initial profile, a precision therapeutic strategy is selected, utilizing either Senolysis (e.g., CAR-T, PROTACs) or Senorejuvenation (partial reprogramming). **D** *Response Monitoring*: Biomarkers including biological age clocks are used to track treatment efficacy, creating a feedback loop to adjust the dose or strategy. This entire process is aimed at the central goal of *Healthspan Extension*. Figure created with BioRender.com.

The future of senotherapy may lie in the integrated, personalized application of these advanced strategies. We envision a hypothetical biomarker-guided clinical workflow (Fig. 4): (1) Patient Stratification: Employ non-invasive biomarkers (see section “The biomarker bottleneck”) to quantify SnC burden and characterize their molecular features, including surface antigens and SASP composition. (2) Tailored Intervention: Select the most appropriate senotherapeutic strategy based on the patient’s SnC profile. For instance, a patient with liver fibrosis and elevated uPAR-expressing SnCs could receive uPAR-targeted CAR-T therapy, whereas a patient with high BCL-xL dependency might benefit from a liver-tropic VHL-based PROTAC. In pulmonary fibrosis, a combination of logic-gated CAR-T cells and ferroptosis inducers could sensitize SnCs to immune-mediated clearance. (3) Synergistic Preconditioning: Implement a 2–4 week high-fiber dietary regimen to modulate the gut microbiome, enhancing hepatic drug uptake and reducing baseline inflammation. This preconditioning may permit the use of lower, safer systemic doses of senolytic agents. (4) Response Monitoring: Continuously track SnC burden and SASP markers to guide dose adjustments and treatment duration, ensuring a durable, effective, and safe therapeutic response. However, this framework remains conceptual, constrained by current biomarker limitations and variability in patient responses.

### The biomarker bottleneck

A major challenge in senotherapy is the absence of reliable, non-invasive biomarkers for quantifying SnC burden in vivo. This limitation hampers the ability to stratify patients, monitor therapeutic responses, and optimize dosing regimens, restricting the clinical translation and personalization of senolytic interventions: (1) PET Imaging: Current strategies include PET imaging using probes that selectively bind senescence-associated markers. For example, probes targeting SA-β-Gal, such as [68Ga]Ga-BGal, have demonstrated efficacy in preclinical models^112,113^. Additionally, radioligands targeting fibroblast activation protein (FAP) are emerging as tools for visualizing senescence-driven fibrosis^114^. (2) Liquid Biopsies: A less invasive and highly promising approach involves analyzing circulating cell-free DNA (cfDNA) from blood samples. SnCs exhibit a distinctive epigenetic landscape, and the cfDNA they release may carry senescence-specific signatures^115^. Techniques such as cfDNA fragmentomics assess fragment sizes and end motifs, while cfDNA methylation profiling can detect epigenetic marks characteristic of senescence and may even indicate the tissue of origin^116^. While measurement of SASP factors, such as IL-6, is common, these markers lack specificity^117^. Therefore, a multi-modal biomarker approach will be crucial for the successful clinical translation of senotherapeutic interventions, though significant development is still required.

## Clinical outlook and translational challenges

### Autoimmune risks and safety monitoring

Immune-based senotherapies inherently carry the risk of immune-related adverse events, including autoimmunity and CRS. These risks may be amplified in older patients due to underlying immune dysregulation associated with “inflammaging.” Implementing rigorous safety protocols, adapted from oncology, such as careful dose escalation and monitoring for autoantibodies, will be essential to ensure patient safety^118,119^.

### Personalized senolysis: the path forward

Individual variability in response to senolytic therapies can arise from sex differences (such as hormonal regulation of drug transporters and immune targets), microbiome composition, and pharmacogenomic factors^120^. For instance, preclinical studies demonstrated that D + Q treatment preserved cognitive function in aging male rats but not in females that had undergone estropause, indicating that hormonal status may modulate senolytic efficacy^121^. Future clinical trials will need to account for these variables to develop personalized dosing and treatment strategies, ensuring maximal efficacy and safety across diverse patient populations^122^.

### Regulatory pathways and cost considerations

Next-generation senolytics face substantial regulatory and economic hurdles. The high production costs of CAR-T cells and PROTACs, reflecting complex manufacturing and purification processes, may limit patient accessibility^123^. Additionally, regulatory bodies such as the FDA currently approve therapies for specific diseases rather than for “aging” itself. A notable initiative addressing this gap is the targeting aging with metformin trial, which seeks to establish a regulatory precedent for aging as a treatable indication by employing a composite endpoint encompassing multiple age-related diseases^124^. For senolytic therapies, establishing validated surrogate endpoints for healthspan and demonstrating long-term safety will be critical, particularly for any prophylactic applications. For PROTACs specifically, additional challenges include resistance mechanisms, limited tissue-specificity of available E3 ligases, poor oral bioavailability, and risks of off-target protein degradation in non-target tissues.

## Future directions and conclusions

### Beyond BCL-xL: artificial intelligence (AI)-driven discovery of the next target frontier

While BCL-family proteins remain central senolytic targets, research is increasingly exploring new vulnerabilities. These include the p21CIP1 pathway^125^, novel SnC surface markers revealed through transcriptomic profiling, and distinctive metabolic dependencies such as glutaminolysis^126^. The discovery of these targets and corresponding drugs is being accelerated by AI. Machine learning pipelines, trained on extensive chemical compound datasets, have already identified novel small-molecule senolytics, such as Ginkgetin, Periplocin, and Oleandrin, and predicted synergistic drug combinations, substantially reducing the time and cost of discovery^127,128^. AI has also facilitated the identification of new targets, including the kinase TNIK, and the design of senomorphic molecules capable of suppressing the SASP^129,130^.

### The evolutionary trade-off: a word of caution

A critical consideration in senotherapy is the evolutionary role of cellular senescence^131^. According to the antagonistic pleiotropy theory, senescence is a conserved mechanism that confers early-life benefits, notably tumor suppression and wound healing, while contributing to age-related decline later in life^132^. Systematically eliminating senescence, especially with persistent “living drugs” like CAR-T cells, may carry unforeseen long-term risks. Preclinical studies have shown that SnC clearance can impair liver regeneration and wound healing under certain conditions, emphasizing the need to differentiate transient, beneficial senescence from chronic, pathological accumulation^133–135^. This duality highlights that transient, targeted interventions may be safer and more effective than permanent, systemic elimination of SnCs^136^.

### Ethical considerations and socioeconomic equity

The development of therapies targeting aging itself raises significant ethical considerations. If such powerful and costly interventions are accessible only to the wealthy, they could create a “longevity dividend”, exacerbating existing social and health inequities. Addressing the high cost of advanced senotherapies and ensuring equitable access will therefore represent a major societal challenge^137,138^.

### Concluding reflections

Senolytic therapy has progressed from a conceptual framework to a sophisticated field at the intersection of geroscience, immunotherapy, and chemical biology^139,140^. While first-generation senolytics established critical proof-of-concept, their inherent limitations—including toxicity, variable efficacy, and resistance—have catalyzed the development of next-generation strategies. As discussed, the future of senotherapy may lie in precision reprogramming through the synergistic modulation of immunity, protein degradation, and metabolism^141,142^. An emerging, conceptually transformative frontier is partial epigenetic reprogramming, which leverages transient expression of Yamanaka factors to rejuvenate cells and reverse senescence markers without inducing cell death, offering a route toward cellular restoration rather than removal^143–145^. Despite formidable challenges, including immunopathology, manufacturing complexity, variable efficacy across models, off-target risks, long-term safety uncertainties, high costs, and ethical concerns, these innovative strategies hold potential to advance the treatment of age-related diseases and extend human healthspan. Moving forward, rigorous, biomarker-guided clinical trials will be essential to realize this potential.

## Data Availability

No datasets were generated or analyzed during the current study.
